# DNA Double-Strand Breaks Are a Critical Regulator of Fear Memory Reconsolidation

**DOI:** 10.3390/ijms21238995

**Published:** 2020-11-26

**Authors:** Shaghayegh Navabpour, Jessie Rogers, Taylor McFadden, Timothy J. Jarome

**Affiliations:** 1 Fralin Biomedical Research Institute, Translational Biology, Medicine & Health, Virginia Polytechnic Institute and State University, Roanoke, VA 24016, USA; navabpour@vt.edu; 2 Department of Biological Sciences, Virginia Polytechnic Institute and State University, Blacksburg, VA 24061, USA; jrogers110@vt.edu; 3 Department of Animal and Poultry Sciences, Virginia Polytechnic Institute and State University, Blacksburg, VA 24061, USA; tmcfadde2020@vt.edu; 4 School of Neuroscience, Virginia Polytechnic Institute and State University, Blacksburg, VA 24061, USA

**Keywords:** epigenetic, histone methylation, DNA double-strand breaks, memory, reconsolidation, retrieval, hippocampus

## Abstract

Numerous studies have shown that following retrieval, a previously consolidated memory requires increased transcriptional regulation in order to be reconsolidated. Previously, it was reported that histone H3 lysine-4 trimethylation (H3K4me3), a marker of active transcription, is increased in the hippocampus after the retrieval of contextual fear memory. However, it is currently unknown how this epigenetic mark is regulated during the reconsolidation process. Furthermore, though recent evidence suggests that neuronal activity triggers DNA double-strand breaks (DSBs) in some early-response genes, it is currently unknown if DSBs contribute to the reconsolidation of a memory following retrieval. Here, using chromatin immunoprecipitation (ChIP) analyses, we report a significant overlap between DSBs and H3K4me3 in area CA1 of the hippocampus during the reconsolidation process. We found an increase in phosphorylation of histone H2A.X at serine 139 (H2A.XpS139), a marker of DSB, in the *Npas4*, but not *c-fos*, promoter region 5 min after retrieval, which correlated with increased H3K4me3 levels, suggesting that the two epigenetic marks may work in concert during the reconsolidation process. Consistent with this, in vivo siRNA-mediated knockdown of topoisomerase II β, the enzyme responsible for DSB, prior to retrieval, reduced *Npas4* promoter-specific H2A.XpS139 and H3K4me3 levels and impaired long-term memory, indicating an indispensable role of DSBs in the memory reconsolidation process. Collectively, our data propose a novel mechanism for memory reconsolidation through increases in epigenetic-mediated transcriptional control via DNA double-strand breaks.

## 1. Introduction

The traditional view of memory storage is that once a memory for a task or learned association is stored or “consolidated,” it becomes stable and no longer susceptible to disruption [1]. This notion has been challenged by various studies showing that previously consolidated memories are susceptible to disruption or modification after retrieval and are re-stabilized through a process called reconsolidation [2,3,4]. This reconsolidation process is controlled by increased transcriptional and translational regulation [4,5,6,7]. In terms of the requirement for de novo gene transcription, several immediate early genes and transcription factors have been implicated in the reconsolidation process [2,8]. For example, the expression of the *Npas4* gene increases after retrieval and is critical for reconsolidation [9,10]. NPAS4 has well-studied roles in learning-dependent synaptic plasticity, such as regulating neuronal plasticity in the hippocampus during contextual learning [11] and linking this neuronal activity with memory [12], and *Npas4* deletion impairs contextual fear memory [10,13,14]. Furthermore, knockdown of Npas4 impairs fear memory reconsolidation [10]. However, it is not clear how the transcription of *Npas4* is regulated during the reconsolidation process.

Recently, several studies have identified a critical role for epigenetic mechanisms in the form of post-translational modification of histone proteins in transcriptional regulation during the reconsolidation process [9,15,16,17]. Histone H3 lysine 4 trimethylation (H3K4me3) is an abundant epigenetic mark that is an essential regulator of gene transcription and a biomarker for active transcription [18,19,20]. In addition to evidence suggesting a role for this histone modification in the consolidation process [21,22], recent evidence indicates that it is also involved in memory reconsolidation. For example, our recent work showed an increase in H3K4me3 at the *Npas4* transcriptional start site in area CA1 of the hippocampus one hour after the retrieval of contextual fear memory, and knockdown of the H3K4me3 enzyme *Mll1* in the hippocampus disrupted the reconsolidation process [9]. However, how H3K4me3 is regulated and coordinated to specific genes, such as *Npas4*, during reconsolidation remains poorly understood.

DNA double-strand break (DSB) is an essential mechanism to solve DNA-related topological problems, such as transcription, chromatin remodeling, and replication, which occurs through the topoisomerase II family, including the topoisomerase IIβ (TOPIIβ) enzyme [23,24]. This enzyme tweaks the supercoiled DNA to facilitate the DNA interaction with proteins [23,25]. Evidence shows that TOPIIβ may open the chromatin structure by catalyzing the transient formation of DSBs followed by an enzymatic rejoining of cleaved DNA ends through its intrinsic intramolecular religation activity [26]. In order to prevent the persistence of unsealed DSBs, and in case of TOPIIβ failure in religation, this process is accompanied by classical DSB repair molecules, such as non-homologous end joining (NHEJ) pathway factors, or possibly homologous recombination (HR) pathway, and other DNA repair pathway proteins [24,27,28]. Furthermore, it has been suggested this enzyme may control neuronal gene transcription by directly binding to their regulatory regions [13,29,30], and that blocking TOPIIβ activity alters the expression of nearly one-third of all developmentally regulated genes [29,31]. Every time that a DSB occurs, the adjacent histone variant H2A.X is phosphorylated at serine 139 (H2A.XpS139) by ataxia telangiectasia mutated (ATM), ATM and Rad3-related proteins (ATR), or DNA-dependent protein kinase complexes (DNA-PKcs), and this marker can be used to investigate the loci and amount of DSBs in genes [32,33,34,35]. Recently evidence has emerged indicating that stimulation of neuronal activity and learning could induce DSBs at actively transcribed genes [13]. Two studies using this biomarker showed considerable overlaps between DNA sites targeted for DSBs and enrichment of H3K4 methylation, mostly in promoter areas [31,36]. However, little is known about the role of DSBs in vivo, and whether DSBs occur following the retrieval of memory remains unknown. Considering the important role of DSBs in neuronal gene transcription, it is critical to understand whether this epigenetic mechanism contributes to the reconsolidation process in order to fully understand the transcriptional processes necessary for memory modification.

Here, we studied the role of DSBs following retrieval of a previously consolidated contextual fear memory and whether it triggers H3K4me3 during the reconsolidation process. Using chromatin immunoprecipitation (ChIP) assays, we found simultaneous increases in H2A.XpS139 and H3K4me3 at the promoter of the *Npas4* gene in the dorsal hippocampus early on in the reconsolidation process. Moreover, our results showed siRNA-mediated knockdown of *TopIIβ* in the dorsal hippocampus prior to retrieval abolished the observed increased in both H2A.X(pS139) and H3K4me3 and impaired fear memory reconsolidation. Collectively, these findings propose a novel mechanism for memory reconsolidation through the increase of gene transcription via DSB early on after memory retrieval.

## 2. Results

### 2.1. Gene-Specific Increases in H2A.XpS139 Levels in the Dorsal Hippocampus Following Memory Retrieval

**Figure 1:**
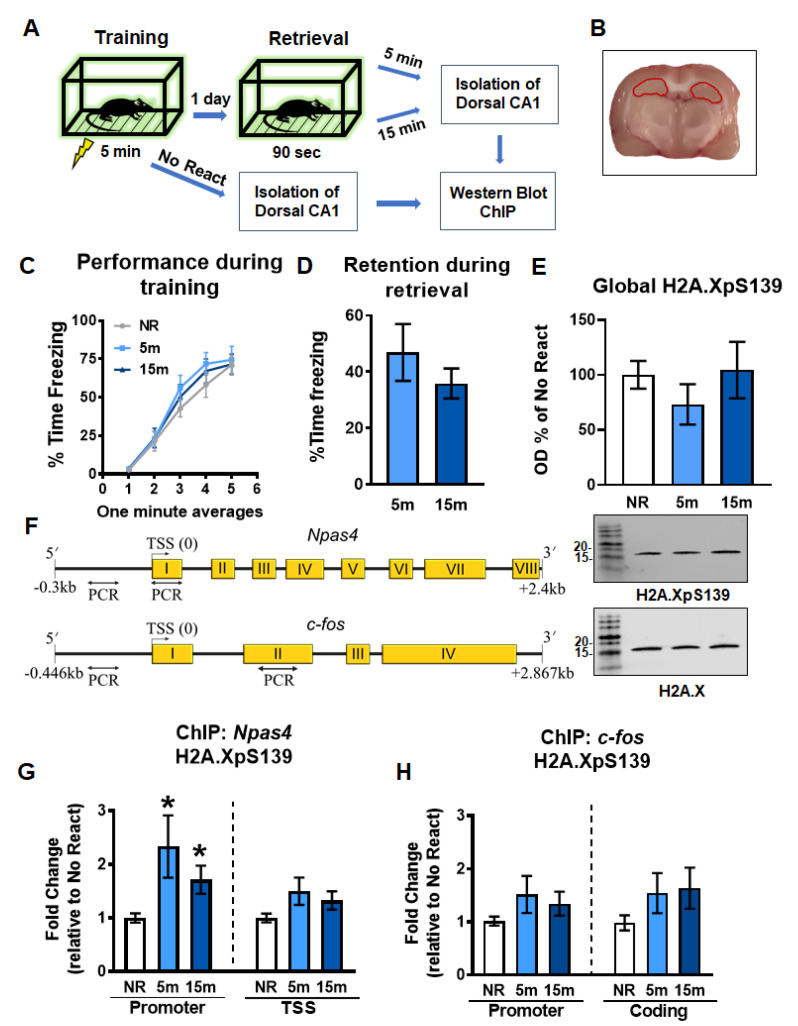
Gene-specific increases in H2A.XpS139 immediately after memory retrieval. (**A**) Experimental design. Rats were trained on a contextual fear conditioning task and exposed to the training context the following day to reactivate the memory. The CA1 region of the dorsal hippocampus was collected 5 or 15 min and processed for western blot and ChIP analyses. (**B**) Image depicting the dorsal hippocampus dissection, which is indicated in red. (**C**) Behavioral performance during the training session. (**D**) Memory retention during the retrieval session. (**E**) Western blot analysis revealed a moderate decrease in H2A.XpS139 levels in bulk histone extracts 5 min after the retrieval, which returned to baseline by 15 min (*n* = 12 per group). Representative bands show H2A.XpS139 (top) and total H2A.X (bottom) from the same gel. (**F**) Schematic showing primer targets for ChIP assays. The promoter and TSS regions of the *Npas4* gene and the promoter and coding region of the *c-fos* gene were targeted. (**G**) ChIP analysis revealed an increase in H2A.XpS139 levels at the *Npas4* promoter, but not TSS, region at 5 and 15 min following retrieval (*n* = 12 per group). (**H**) No changes in H2A.XpS139 were observed in either promoter or coding region of the *c-fos* gene following retrieval (*n* = 12 per group). NR: No React, TSS: Transcriptional start site. * Denotes *p* < 0.05 from No React.

Transcriptional regulation and de novo protein synthesis have been shown to be crucial for the reconsolidation of contextual fear memory in the hippocampus [6,9,37]. Madabhushi et al. [13] showed conditioning paradigm. However, whether DSBs are potentially involved in transcriptional regulation critical for memory reconsolidation has yet to be elucidated. To test this in reconsolidation, we first assessed global and gene-specific H2A.XpS139 levels in the dorsal hippocampus 5 and 15 min following retrieval of a contextual fear memory using western blotting and ChIP assays (Figure 1A–D). In bulk histone extracts from the CA1 region of the dorsal hippocampus, we did not observe any significant changes in H2A.XpS139 levels (W_(2.000, 17.00)_ = 0.8075, *p* = 0.4624; Figure 1E). Since H2A.XpS139 levels could be changing in a gene-specific manner, which may not be detected in bulk histone extracts, we next examined levels of this histone modification at the *Npas4* and *c-fos* genes (Figure 1F), two well-described regulators of memory reconsolidation that undergo extensive epigenetic editing following retrieval [10,35]. ChIP analyses revealed a significant increase in H2A.XpS139 levels within the *Npas4* gene promoter (W_(2.000, 16.39)_ = 5.446, *p* = 0.0154), but not transcriptional start site (TSS; W_(2.000, 17.48)_) = 2.736, *p* = 0.0926), at 5 and 15 min after retrieval relative to the no retrieval control (Figure 1G). However, this was not the case for the *c-fos* gene where H2A.XpS139 levels did not change in the promoter (W_(2.000, 15.60)_ = 1.690, *p* = 0.2166) or coding (W_(2.000, 17.08)_ = 1.891, *p* = 0.1813) regions (Figure 1H). Collectively, these data suggest memory retrieval triggers gene-specific DSBs within promoter regions early on in the reconsolidation process, and that DSBs increased in the hippocampus 15 min after training in the contextual fear.

### 2.2. Gene-Specific H3K4me3 Levels Increase Simultaneously with H2A.XpS139 in the Dorsal Hippocampus Following Retrieval

**Figure 2:**
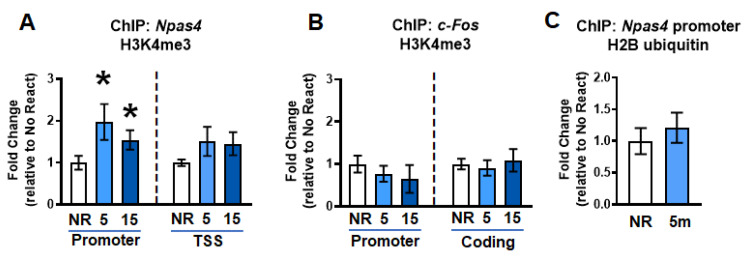
Gene-specific increases in H3K4me3 immediately after memory retrieval. (**A**) ChIP analysis showed an increase in histone H3 lysine 4 trimethylation (H3K4me3) levels at the *Npas4* promoter, but not TSS, region at 5 min after retrieval compared to the no react (NR) group (*n* = 12 per group). (**B**) There were no changes in H3K4me3 levels in *c-fos* promoter or coding region 5 or 15 min after retrieval (*n* = 12 per group). (**C**) No changes were observed in monoubiquitination of histone H2B (H2Bubi) at the *Npas4* promoter following retrieval. * Denotes *p* < 0.05 from No React.

DSBs have been reported to be associated with the accumulation of H3K4me3, which has been implicated in transcriptional control during memory reconsolidation [9]. We next tested whether changes in H3K4me3, a transcriptional activator, occurs at the same genes with increased H2A.XpS139 following retrieval. Results did not show a main effect for group for changes in H3K4me3 levels within the *Npas4* promoter (W_(2.000, 18.27)_ = 3.259, *p* = 0.0616) or coding region (W_(2.000, 14.97)_ = 2.055, *p* = 0.1627) 5 min after retrieval (Figure 2A). To further investigate whether there were time-dependent changes in H3K4me3 binding at the *Npas4* promoter, which is possible considering that histone methylation changes can be transient, we ran pairwise *t*-tests for both 5 and 15 min with NR group. Based on this analysis, there was a significant increase in both 5 (*t*_13.00_ = 2.120, *p* = 0.0269) and 15 (*t*_18.33_ = 1.925, *p* = 0.0349) minutes compared to No React group. This pattern is similar to that of H2A.XpS139 mark, suggesting H3K4me3 may target the same genes as DSBs immediately following retrieval. Consistent with this, like H2A.XpS139, H3K4me3 levels did not change in the *c-fos* promoter (W_(2.000, 18.57)_ = 0.5277, *p* = 0.5986) or coding (W_(2.000, 18.42)_ = 0.1672, *p* = 0.8473) regions following retrieval (Figure 2B). Furthermore, we did not observe an increase in monoubiquitination of histone H2B at lysine 120 (H2Bubi; *t*_18.31_ = 0.6768, *p* = 0.2535; Figure 2C), a marker of active transcription that is often associated with H3K4me3, suggesting that DSBs are associated with some, but not all, epigenetic marks following retrieval. Importantly, this overlap between H2A.XpS139 and H3K4me3 following retrieval suggests that they may work in concert to regulate the transcription of some, but not all genes, during the reconsolidation process.

### 2.3. Knockdown of TopIIβ in the Dorsal Hippocampus Abolishes Retrieval-Induced Increases in Gene-Specific H2A.XpS139 and H3K4me3

**Figure 3:**
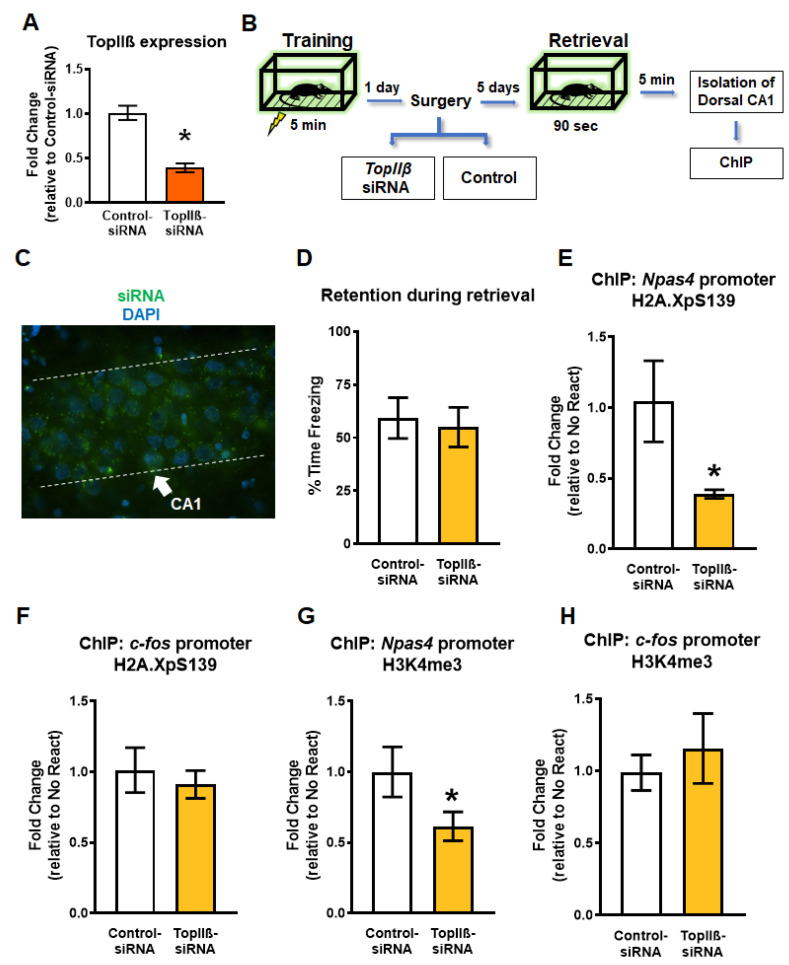
Knockdown of the topoisomerase enzyme producing DSB (*TopIIβ*) in the dorsal hippocampus prior to retrieval reduces gene-specific H2A.XpS139 and H3K4me3 levels. (**A**) In vitro confirmation of successful *TopIIβ* knockdown using Accell siRNA in rat B35 cell culture (*n* = 4 per group). (**B**) Experimental design. Rats are trained on a contextual fear conditioning task and the next day received stereotaxic infusion of Accell siRNAs targeting *TopIIβ* or a scrambled sequence (Control-siRNA). Five days after infusion, animals were re-exposed to the training context and the CA1 region of the dorsal hippocampus was collected 5 min later. (**C**) Fluorescent microscopy image showing control green siRNA clusters in the dorsal CA1 region. DAPI was used to visualize nuclei. (**D**) There were no differences in memory retention during the retrieval session (*n* = 9 per group). (**E**,**G**) ChIP analysis revealed a significant reduction in both H2A.XpS139 and H3K4me3 levels at the *Npas4* gene promoter 5 min after retrieval in animals that received the siRNA against *TopIIβ* prior to retrieval. (**F**,**H**) Knocking down *TopIIβ* prior to retrieval did not alter H2A.XpS139 or H3K4me3 levels at the *c-fos* gene promoter 5 min after retrieval. * Denotes *p* < 0.05 from Control-siRNA.

The results described above indicated an association between DSBs (H2A.XpS139) and H3K4me3 at specific learning-permissive genes following retrieval. Some evidence suggests that DSBs may lead to H3K4me3 increase by recruiting its methyltransferases, MLL1 [38]; however, it is unknown if this occurs in the brain following memory retrieval. In order to test this, we next performed in vivo siRNA experiments targeting *TopIIβ*. In order to do this, we used an Accell siRNA against *TopIIβ*, which we confirmed was successful at reducing gene expression in rat B35 cells (*t*_4.952_ = 6.495, *p* = 0.0013; Figure 3A). Next, animals were trained to the fear conditioning task followed by a hippocampal-infusion of the same Accell siRNA targeting the DSB enzyme gene *TopIIβ* (TopIIβ-siRNA), or a control scrambled siRNA sequence (Control-siRNA), 24 h later (Figure 3B). These siRNAs are highly specific with a fast induction rate in vivo (Figure 3C), which results in a transient and reversible knock down in gene expression [9,17,39]. Five days after siRNA infusion, the rats were re-exposed to the training context for 90 s (Figure 3D), and the dorsal CA1 region was collected 5 min later for ChIP analysis. We found the knockdown of *TopIIβ* resulted in a significant reduction in both H2A.XpS139 (U = 16.50, *p* = 0.0311; Figure 3E) and H3K4me3 (*t*_12.59_ = 1.880, *p* = 0.0417; Figure 3G) levels at the *Npas4* promoter region relative to the control group. Importantly, levels of H2A.XpS139 (*t*_11.84_ = 0.5441, *p* = 0.5695; Figure 3F) or H3K4me3 (*t*_11.77_ = 0.6186, *p* = 0.2740; Figure 3H) did not change at the *c-fos* promoter region following retrieval. Taken together, these findings suggest that H2A.XpS139 is an important regulator of retrieval-induced increases of H3K4me3 in the *Npas4* promoter.

### 2.4. Knockdown of TopIIβ in the Dorsal Hippocampus Prior to Retrieval Impairs Memory Reconsolidation

**Figure 4:**
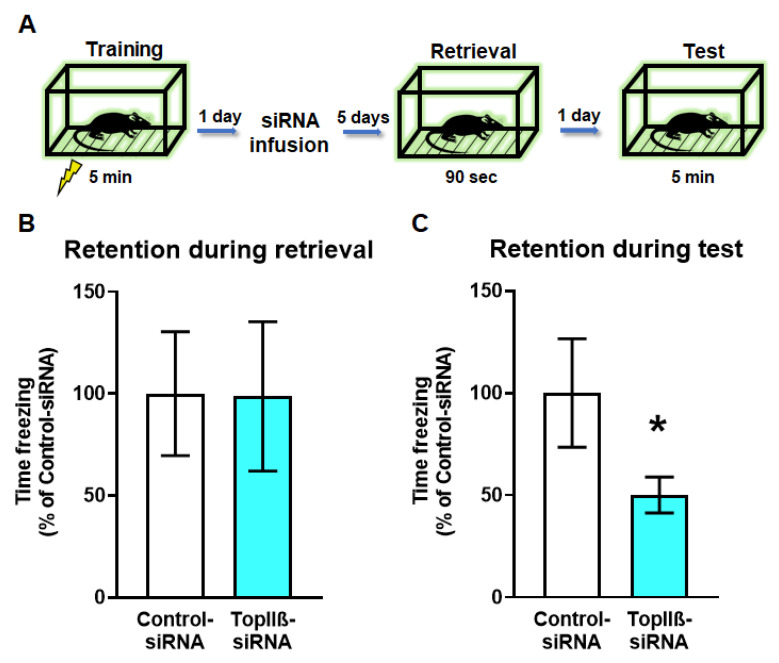
Knockdown of the topoisomerase enzyme producing DSB (*TopIIβ*) in the dorsal hippocampus prior to retrieval impairs memory reconsolidation. (**A**) Experimental design. Rats are trained on a contextual fear conditioning task and the next day received stereotaxic infusion of Accell siRNAs targeting *TopIIβ* or a scrambled sequence (Control-siRNA). Five days after infusion, animals were re-exposed to the training context to reactivate the memory and tested the following day. (**B**,**C**) There were no differences in memory retention during the retrieval session (**B**; n = 9 per group), but (**C**) *TopIIβ* knockdown significantly impaired memory the following day. * Denotes *p* < 0.05 from Control-siRNA.

So far, we have shown that DSBs and H3K4me3 are upregulated within the *Npas4* promoter region early on in the reconsolidation process and that loss of H2A.XpS139 reduces H3K4me3 levels following the retrieval. However, it is currently unknown whether DSBs are necessary for memory reconsolidation. To address this, we trained rats to contextual fear conditioning followed by siRNA (TopIIβ-siRNA or Control-siRNA) delivery into the dorsal hippocampus 24 h later (Figure 4A). Five days after siRNA infusion, the rats were re-exposed to the training chamber to reactivate the memory, and twenty-four hours later were placed back into the training chamber to test retention for the task. Our results showed that both groups showed similar fear to the context during retrieval (*t*_15.79_ = 0.2689, *p* = 0.7915; Figure 4B), suggesting that the siRNA manipulation did not impact the ability of the animals to retrieve the memory. However, downregulation of *TopIIβ* resulted in a significant deficit in their memory retention for the context during the final test (U = 13.0, *p* = 0.0499; Figure 4C), indicating blockade of the reconsolidation process. Collectively, these data show that DSBs are a critical regulator of memory reconsolidation, which may occur via recruitment of H3K4me3 to specific learning-permissive genes following retrieval.

## 3. Discussion

There is strong evidence that epigenetic mechanisms regulate the transcription of genes necessary for memory consolidation in several brain regions [16,40,41,42,43]. However, very little is known about the role of epigenetic mechanisms in the reconsolidation process. In the present study, we investigated the role of DSBs in the reconsolidation of a fear memory and in controlling retrieval-dependent H3K4me3 in the dorsal hippocampus. Our results show both H2A.XpS139 and H3K4me3 are upregulated at the promoter of the *Npas4* gene early in the reconsolidation process in the dorsal hippocampus. Further, we found that knocking down the enzyme hydrolyzing DSBs prior to retrieval reduced H3K4me3 and impaired long-term memory. Collectively, these findings propose a novel mechanism for memory reconsolidation through the increase of gene transcription via DSBs early on after memory retrieval.

Several studies have shown that neuronal activity caused by exploring a novel environment or neuronal stimulation with NMDA may trigger DSB formation in vitro and in vivo [13,44,45,46]. Recently, two independent studies have presented DSB as an important factor involved in fear memory consolidation, which in turn increases the expression of a subset of IEGs. Results from Li et al. [47] study showed that DSBs are induced in response to cued fear learning in the promoter area of some IEGs, such as *c-Fos, Arc*, and *Npas4*, during memory consolidation, resulting in an increase in their transcription levels. Similarly, Madabhushi et al. [13] detected elevated levels of H2A.XpS139 in the hippocampus of mice trained on a contextual fear conditioning task. However, the importance of DSB during the reconsolidation process has remained unexplored. In the present study, we found that H2A.XpS139 levels were increased in the hippocampus immediately following memory retrieval, and downregulating *TopIIβ* prior to the retrieval prevented this increase and impaired fear memory, providing the first evidence that in addition to memory consolidation, DSBs play a critical role in the reconsolidation process.

A recent study revealed that the binding levels of H3K4me3 to the *Npas4* gene transcriptional start site were increased in the dorsal hippocampus 1 h after retrieval of a contextual fear memory [9]. They also found that blocking the methyltransferase Mll1 prior to the retrieval prevents these increases and impaired memory [9]. Furthermore, H3K4me3 levels have been shown to be increased in the CA1 area during the consolidation of a contextual fear memory [48]. Our results are consistent with these results and extend them by showing that an elevation of H3K4me3 levels occurs at the *Npas4* promoter in the dorsal hippocampus 5 and 15 min after memory retrieval, suggesting this histone methylation mark may increase in expression more rapidly following behavioral training than previously thought. While it is unclear why *Npas4* H3K4me3 changes occurred in different regions (promoter vs TSS) at different time points after retrieval, such a result is possible considering that histone methylation can be transient, particularly in cases in which nucleosome remodeling is initiated, for example by binding of TIP60. This could explain why we observed increases in H3K4me3 in the *Npas4* promoter 5 and 15 min after retrieval in the present study, but not at 1 h in the previous work [9]. Future studies should explore in more detail the temporal dynamics of H3K4me3 levels and gene expression changes following memory retrieval.

Numerous in vivo and in vitro studies have demonstrated that topoisomerases have a prominent role in transcriptional regulation. Based on these studies, when gene enhancers recruit topoisomerases, it will release the torsional stress of the chromatin more frequently and maintain the open chromatin state, which will increase the frequency of transcription of that gene to achieve different expression levels [49,50,51,52]. This effect is believed to be through topoisomerases managing the positive and negative DNA supercoiling that accumulates ahead of and behind RNA polymerase II (PolII), respectively [53,54]. Further, some believe this enzyme family is also necessary for preserving the negative supercoiling required for initiating gene transcription at the promoter area [55,56]. Moreover, inhibition of TOPIIβ or TOPI, a topoisomerase that creates single-strand DNA breaks, leads to a decrease in the transcription of long genes [56,57], and the repression of the basal transcription levels [50], respectively. Interestingly, Tiwari et al. [31] showed a strong relationship between target sites of TOPIIβ and chromosome regions containing H3K4 methylation marks. Similarly, Husain et al. [58] were able to partially replicate their results and showed that chromatin sites associated with TOPI are also enriched in H3K4 methylation. These observations are consistent with our present study in which we observed co-localization of H2A.XpS139 and H3K4me3 soon after retrieval at the *Npas4* gene promoter, suggesting that these two epigenetic mechanisms may work in concert to regulate the transcription of the *Npas4* gene during reconsolidation. Based on our results, it would be interesting to speculate that memory retrieval triggers the transcription of genes necessary for reconsolidation via the TOPIIβ enzyme relaxing the chromatin structure. This would, in turn, initiate and maintain the transcription of critical genes, such as *Npas4*, by producing DSBs in the promoter region, resulting in the recruitment of H3K4me3 to regulate active transcription. However, this mechanism of transcriptional control may not occur for all memory-permissive genes, as we surprisingly found that this did not occur at *c-fos*. Future studies are needed then to validate this hypothesis and determine what the gene targets are for the DSBs recruitment of H3K4me3 during the reconsolidation process.

Our data strongly suggest a role for DSBs in the reconsolidation process following retrieval. However, there are several limitations to the present study that need to be considered when interpreting our data. First, memory storage process often has prolonged or multiple phases of transcriptional regulation [59]. While we observed rapid increases in DSBs at 5 min after retrieval, our approach did not allow us to assess if these DSB marks are persistent or biphasic. Another concern is our siRNA approach, while highly specific, results in persistent reductions in gene expression. It is possible that DSB levels could change during other stages of memory storage, such as maintenance or retrieval, which could affect memory independent of reconsolidation [9]. However, our siRNA approach lacks the temporal control needed to separate reconsolidation effects from those of offline maintenance or retrieval. Future studies should aim to use more temporally controlled manipulations of DSBs to assess the exact contribution of this mechanism to the reconsolidation process.

In summary, we present the first evidence that DNA double-strand breaks are a likely regulator of the reconsolidation process. Importantly, our data suggest that DSBs may act as an epigenetic initiator following memory retrieval. These results add to the rapidly expanding understanding of the molecular mechanisms involved in memory reconsolidation and the role of epigenetic modifications in this process.

## 4. Materials and Methods

### 4.1. Animals (Subjects)

A total of 75 male 8–9 weeks old Sprague Dawley rats (Envigo, Indianapolis, IN, USA) weighing 250–300 g at the time of arrival were used in these experiments. Rats were housed two per cage with free access to water and rat chow, and were maintained on a 12:12 h light:dark cycle. All procedures were approved by the Virginia Polytechnic Institute and State University Institutional Animal Care and Use Committee (Protocol #18-019, approval 3/31/2018) and conducted within the ethical guidelines of the National Institutes of Health.

### 4.2. Surgery and siRNA Delivery

Rats bilaterally were injected with Accell siRNAs (Dharmacon, Lafayette, CO, USA) into their dorsal CA1 of the hippocampus using stereotaxic coordinates (AP −3.6 mm, ML ± 1.7 mm, DV −3.6 mm) relative to bregma as described previously with a small scale modification [9,17,36]. Animals were anesthetized with 2–4% isoflurane and the infusion was given over a 10 min period (0.1 µL per minute) for a total volume of 1 µL per hemisphere. Animals were left in their home cage to recover for 5 days before retrieval. Fresh Accell SMARTpool *TopIIβ* (#E-087922-01-0005) and nontargeting control (#D-001910-10-05) siRNA stocks (100 µM) were resuspended in Acell siRNA delivery media (#B-005000-100) to a concentration of ~4.5 µM on the day of surgery. A nontargeting green fluorescent Accell siRNA (#D-001950-01-05) was used to confirm the targeting of the dorsal CA1 region.

### 4.3. Cell Culture

Rat B35 neuroblastoma cell line (#CRL-2754; ATCC, Manassas, VA, USA) were cultured in Dulbecco’s Modified Eagle’s Medium (DMEM) (#30-2002; ATCC, Manassa, VA, USA) supplemented with 10% Fetal Bovine serum (#35-016-CV; Corning, Tewsbury, MA, USA) and 0.1% Penicillin/Streptomycin (#15070063; Gibco, Gaitherburg, MD, USA). One day prior to transfection, cells at 70–90% confluency in a 100 mm dish were treated with 0.05% Trypsin-EDTA (1X) (#25300054; Gibco, Gaitherburg, MD, USA). Cells were then placed into a 96-well dish with a 1:56 ratio of cells going into each well containing 100 µL of DMEM-based media. Transfection was conducted using Accell siRNAs following manufacturer’s instructions. Briefly, on the day of transfection, DMEM-based media was removed and cells were washed with DPBS (#14190144; Gibco, Gautherburg, MD, USA). In a separate tube, 7.5 μL of the 100 μM siRNA was mixed with 750 μL Accell Delivery Media (Cat #B-005000, Dharmacon, Lafayette, CO, USA). The final concentration was 1 μM Accell siRNA per well in a 96-well plate. Sixteen wells were transfected with the scrambled siRNA (control) and sixteen wells were transfected with TopIIβ-siRNA. Cells were cultured in a NAPCO series 8000 Water Jacket CO_2_ incubator (model 3586, Thermo Fisher, Waltham, MA, USA) for 48 h post transfection. Every 4 wells of each group were combined together to make n = 4/group. Then, RNA was isolated from the cells by TRIzol (#15596018; Ambion, Austin, TX, USA) following the manufacturer’s instructions. cDNA synthesis and real-time PCR were performed as described below.

### 4.4. cDNA Synthesis and Quantitative Real-Time PCR

RNA concentration was measure on the Take3 (BioTek, Winooski, VT, USA), normalized (200 ng), and converted to cDNA using the iScript cDNA synthesis kit (Bio-rad, Hercules, CA, USA). Real-time PCR amplifications of the cDNA were performed on the Bio-rad CFX96 Real-Time System using the following protocol: 95.0 °C for 3 min, then 95.0 °C for 10 s, followed by 60 °C for 30 s (39 repeats), 55–95 °C for 0.5 °C/cycle, followed by a melt curve starting at 55.0 °C for 10 s (81 repeats), and then held at 4.0 °C. Primers were: *TopIIβ* (F: TGGTTTACGGAAGGAGTGGC; R: CGCAGCCTTTTCTTGTGCTT) and Gapdh (F: ACCTTTGATGCTGGGGCTGGC; R: GGGCTGAGTTGGGATGGGGACT) used as an internal control and data was analyzed using the comparative Ct method.

### 4.5. Apparatus

The Habitest chamber (#H10-11R-TC, Calbourne, Holliston, MA, USA) consisted of a steel test cage with front and back Plexiglas walls and a grid shock floor above a plastic drop pan. The right wall of the chamber consisted of a house light in the top back corner, which remained on during the behavioral procedures, and an infrared light in the top middle, which was not illuminated during this project. The left wall of the chamber consisted of a high-bright light, which was not illuminated during this project. All remaining slots of both walls were filled with blank metal panels. A USB camera was mounted on a steel panel outside the back Plexiglas wall of the chamber, angled at ~45 degrees. The entire chamber was housed in an isolation cubicle with an acoustic liner and a house fan, which remained active during all behavioral procedures. Shock was delivered through the grid floor via a Precision Animal Shocker under the control of FreezeFrame 4 software (#AM1-FF04, Calbourne, Holliston, MA, USA, which also analyzed animal behavior in real-time. All video was recorded and stored for later analysis.

### 4.6. Behavioral Procedures

The contextual fear conditioning procedure was performed as described previously in Orsi et al. [60]. Briefly, animals were handled for two days followed by two days of acclimation to the transport procedure before the start of behavioral training. Rats were then trained to a standard contextual fear-conditioning paradigm in which four shock presentations (1.0 mA, 2 s, 60 s ITI) were given over a 5 min session in a novel context. Following the completion of training, rats were returned to their home cages. To reactivate the memory, rats were placed back into the training context for 90 s in the absence of any foot shocks; no retrieval rats underwent the same training protocol but were not re-exposed to the training context on the retrieval day. Testing occurred the following day and consisted of a 5 min exposure to the training context. Due to variability in behavioral performance across cohorts, experimental animals were normalized to the control group for that specific cohort for retrieval and testing sessions.

### 4.7. Tissue Collection and Protein Extraction

Animals were overdosed on isoflurane in a necrosis chamber and the brains were rapidly collected and immediately frozen on dry ice. Tissue containing the CA1 region of the dorsal hippocampus were dissected on dry ice with the aid of a rat brain matrix. The hemispheres were split so that each one was used for different analyses, which was counterbalanced across conditions to account for any potential laterality effects. All dissected tissues were frozen at −80 °C until needed.

### 4.8. Histone Extraction

Histone extractions were performed as described previously [17]. Briefly, tissues were homogenized in non-denaturing sucrose buffer and subjected to centrifugation at 7700× *g* for 1 min. The pellet containing nuclei was resuspended in 250 µL of 0.4N sulfuric acid, and then incubated on ice for 30 min followed by centrifugation at 14,000× *g* for 30 min at 4 °C. The resulting supernatant was mixed with trichloroacetic acid with 4 mg/mL deoxycholic acid and incubated on ice for 30 min. Precipitated protein was recovered by centrifugation and followed by acetone drying. All procedures were carried out under ice-cold conditions. The purified histone enriched protein pellet was resuspended in 10 mM Tris (pH 8.0). Protein concentrations were determined by the Bradford assay (Bio-rad, Hercules, CA, USA).

### 4.9. Antibodies

Antibodies included phosphorylated H2A.X at serine 139 (1:500 for western blot, 5 µg for ChIP; #05-636; MilliporeSigma, Burlington, MA, USA), trimethylated H3 lysine 4 (5 µg for ChIP; #04-745, MilliporeSigma, Burlington, MA, USA) and total H2A.X (1:1000 for western blot; #2595S, Cell Signaling, Danvers, MA, USA).

### 4.10. Western Blotting

Normalized samples (3 µg) were loaded on 20% Acrylamide gels, ran through SDS-PAGE and transferred onto an Immobilon-FL membrane using a Turbo Transfer System (Biorad, Hercules, CA, USA). Membranes were then incubated in a 50:50 LI-COR blocking buffer (50% LI-COR TBS blocking buffer and 50% TBS + 0.1% Tween-20) for 1 h at room temperature, following by overnight incubation in primary antibody at 4 °C. After three washes with TBS + 0.1% Tween-20 (TBSt) for 10 min, the membranes were incubated in secondary antibody (1:20000; LI-COR) for 45 min. Subsequently, the membranes were washed thoroughly in TBSt for 10 min twice, and once in TBS before imaging them using the Odyssey Fc near infrared system (LiCOR, Lincoln, NE, USA). Image Studio Ver 5.2. was used to quantify proteins. Each sample was normalized to H3, which was used as a loading control; total H2A was not used as a loading control due to it being a target of ubiquitination, which results in variable levels that are unrelated to protein loading. After each development, membranes were stripped for 10 min with 0.2 NaOH followed by two 15 min washes in TBSt and blocking buffer for 1 h.

### 4.11. Chromatin Immunoprecipitation (ChIP)

ChIP assays were performed as described previously with a small scale modification [17]. The dorsal CA1 region of hippocampus was dissected in ice-cold PBS solution containing protease and phosphatase inhibitors and then fixed in PBS with 1% formaldehyde at 37 °C for 10 min. Then, the tissues were washed with ice-cold PBS five times, homogenized in hypotonic buffer (10 mM KCl, 20 mM HEPES, 1 mM MgCl, 1 mM DTT) containing protease and phosphatase inhibitors and centrifuged at 1350× *g* for 10 min at 4 °C to pellet nuclei. The resulting pellet was resuspended in ChIP sonication buffer (1x Tris-EDTA (TE) buffer with 1% SDS) with protease inhibitor and DNA sheared to ~300 bp using the QSonic 800R2 Sonicator with 70% amplitude and 20 s pulse for 30 min. Lysates were centrifuged at 20,000× *g* for 10 min at 4 °C to pellet debris and DNA content was measured using a nanodrop or Take3. Normalized amounts of DNA were diluted in TE buffer and 2x RIPA buffer (2x PBS, 1% sodium deoxycholate, 2% NP-40, 0.2% SDS) with 2x protease inhibitor. Samples were added to Magna ChIP protein A/G magnetic beads (MilliporeSigma, Burlington, MA, USA) incubated with antibodies (anti-H2A.XpS139 or anti-H3K4me3) or no antibody (control) overnight at 4 °C. Immune complexes were sequentially washed with low-salt buffer (20 mm Tris, pH 8.0, 0.1% SDS, 1% Triton X-100, 2 mm EDTA, 150 mm NaCl), high-salt buffer (20 mm Tris, pH 8.1, 0.1% SDS, 1% Triton X-100, 500 mm NaCl, 1 mm EDTA), LiCl immune complex buffer (0.25 M LiCl, 10 mm Tris, pH 8.1, 1% deoxycholic acid, 1% IGEPAL-CA 630, 500 mm NaCl, 2 mm EDTA), and twice with TE buffer, each for three minutes. Immune complexes were eluted with TE buffer containing sodium bicarbonate and 1% SDS. The eluted DNAs were heated at 65 °C overnight to revert the DNA-protein cross-links. DNA was digested by proteinase K (100 µg, 2 h at 37 °C), extracted by phenol chloroform, precipitated by ethanol and subject to quantitative real-time PCR (qRT-PCR) using primers specific to the rat *Npas4* and *c-fos* promoters or coding regions, which have been previously described [9]. PCR amplification occurred on the CFX96 real-time machine (Bio-rad, Hercules, CA, USA) with the following parameters: 95 °C for 3 min, 50 repeats of 95 °C for 10 s followed by 30 s at 60 °C, and 95 °C for 1 min.

### 4.12. Statistical Analyses

All data presented as mean with standard error. Any data point that was two or more standard deviations from the mean was considered as an outlier. The number of outliers, the figure they were identified on and the grouped they were from are as follows: 1E (NR: 1, 5 m: 2, 15 m: 1), 1G (15 m-TSS: 1), 1H (5 m-Promoter: 1, 15 m-Promoter: 1, 15 m-Coding: 1), 2A (TSS groups each had one outlier), 2B (all groups had one outlier except for the NR-Promoter), 2C (NR: 1, 5 m: 2), 3F (Control-siRNA: 1), 3G (TopIIβ-siRNA: 1), 3H (Control-siRNA: 1), 4B (TopIIβ-siRNA: 1), and 4C (one from each group). H2A.XpS139 and behavioral experiments with two groups were analyzed using two-tailed Welch independent samples *t*-test for parametric data or Mann–Whitney test for nonparametric data. Three group H2A.XpS139 experiments were compared with Welch’s Analysis of Variance (ANOVA) and unpaired t with Welch correction post hoc test. Due to a priori hypotheses regarding the relationship between H2A.XpS139 and histone methylation, all H3K4me3 analyses used one-tailed Welch independent samples *t*-tests.
